# A lung cancer risk warning model based on tongue images

**DOI:** 10.3389/fphys.2023.1154294

**Published:** 2023-06-01

**Authors:** Yulin Shi, Dandan Guo, Yi Chun, Jiayi Liu, Lingshuang Liu, Liping Tu, Jiatuo Xu

**Affiliations:** ^1^ Experimental Education Center of Shanghai University of Traditional Chinese Medicine, Shanghai, China; ^2^ School of Basic Medicine, Shanghai University of Traditional Chinese Medicine, Shanghai, China; ^3^ Longhua Hospital Affiliated to Shanghai University of Traditional Chinese Medicine, Shanghai, China

**Keywords:** benign pulmonary nodule, lung cancer, tongue image, machine learning, risk warning model

## Abstract

**Objective:** To investigate the tongue image features of patients with lung cancer and benign pulmonary nodules and to construct a lung cancer risk warning model using machine learning methods.

**Methods:** From July 2020 to March 2022, we collected 862 participants including 263 patients with lung cancer, 292 patients with benign pulmonary nodules, and 307 healthy subjects. The TFDA-1 digital tongue diagnosis instrument was used to capture tongue images, using feature extraction technology to obtain the index of the tongue images. The statistical characteristics and correlations of the tongue index were analyzed, and six machine learning algorithms were used to build prediction models of lung cancer based on different data sets.

**Results:** Patients with benign pulmonary nodules had different statistical characteristics and correlations of tongue image data than patients with lung cancer. Among the models based on tongue image data, the random forest prediction model performed the best, with a model accuracy of 0.679 ± 0.048 and an AUC of 0.752 ± 0.051. The accuracy for the logistic regression, decision tree, SVM, random forest, neural network, and naïve bayes models based on both the baseline and tongue image data were 0.760 ± 0.021, 0.764 ± 0.043, 0.774 ± 0.029, 0.770 ± 0.050, 0.762 ± 0.059, and 0.709 ± 0.052, respectively, while the corresponding AUCs were 0.808 ± 0.031, 0.764 ± 0.033, 0.755 ± 0.027, 0.804 ± 0.029, 0.777 ± 0.044, and 0.795 ± 0.039, respectively.

**Conclusion:** The tongue diagnosis data under the guidance of traditional Chinese medicine diagnostic theory was useful. The performance of models built on tongue image and baseline data was superior to that of the models built using only the tongue image data or the baseline data. Adding objective tongue image data to baseline data can significantly improve the efficacy of lung cancer prediction models.

## 1 Introduction

According to the World Health Organization’s International Agency for Research on Cancer (IARC) global burden of cancer data, there were 19.3 million new cancer cases globally in 2020, of which 2.2 million (11.4%) were new cases of lung cancer, ranking second among all cancers worldwide; lung cancer is still the leading cause of cancer death, accounting for 1.8 million deaths (18%) (Siegel et al., 2021; Sung et al., 2021) and is also the most common type of cancer in China (Cao et al., 2021). Because there are no obvious clinical symptoms of early-stage lung cancer, most patients are in an advanced stage when they are diagnosed, causing them to miss the best treatment period. The majority of advanced lung cancer patients live for less than a year, especially those in high-risk groups.

Pulmonary nodules are one of the most common types of lung pathology (Mazzone and Lam, 2022), and malignant pulmonary nodules are one of the most common early-stage manifestations of lung cancer. Early detection of lung cancer can be achieved through accurate diagnosis of the type and nature of pulmonary nodules and is therefore of great clinical importance. Early detection of benign and malignant pulmonary nodules is critical for improving patient survival and lung cancer prognoses, and reducing overdiagnosis and treatment of patients with benign pulmonary nodules. With the widespread use of high-resolution multislice spiral computed tomography (CT) in recent years, the detection rate for pulmonary nodules has gradually increased. For lung cancer screening, low-dose CT is commonly used. The National Lung Screening Trial found that screening with low-dose CT reduced lung cancer mortality by 20%, and one trial (NELSON) found that low-dose CT screening had a sensitivity and specificity of 85% and 99%, respectively (Horeweg et al., 2014). A Japanese study found that low-dose CT screening for lung cancer is more sensitive than routine chest X-ray screening, but it had a lower specificity and was associated with the possibility of overdiagnosis (Toyoda et al., 2008). Bronchoscopy and biopsy are the gold standards for diagnosing lung cancer. Although bronchoscopy is a minimally invasive technique, it still causes discomfort in patients, is expensive, and has the potential to cause complications, particularly when biopsies are performed on suspicious tissues. Although significant progress has been made in the early detection of lung cancer in recent years, early detection is still inaccurate. The majority of techniques and methods currently in use cannot effectively avoid a diagnosis of advanced stage lung cancer, and so the early detection of lung cancer remains difficult (Mohan et al., 2020; Nightingale et al., 2023).

Tongue diagnosis is a method for comprehensively evaluating the functional state of the body based on an assessment of the tongue. According to studies, tongue images are more accurate than blood biomarkers in detecting gastric cancer, and tongue diagnosis can be used as a stable method for gastric cancer diagnosis (Yuan et al., 2023). The condition of the gastric mucosa can be predicted by tongue images in patients with chronic gastritis (Shang et al., 2022). With the development of the four traditional Chinese medicine (TCM) diagnostic information technologies, various tongue diagnosis instruments have been widely used in clinical practice, the standardized acquisition and analysis of objective data for tongue diagnosis has gradually matured (Jiang et al., 2021a; Li et al., 2021a). Key intelligent tongue diagnosis technologies include tongue image acquisition systems, tongue body segmentation technologies, tongue body and tongue coating separation systems, and feature extraction systems. Image correction, image denoising, tongue body segmentation, tongue body and tongue coating segmentation are performed on the collected tongue images, and then the color and morphological properties of the tongue body and tongue coating are analyzed and summarized (Wang et al., 2013; Xu et al., 2020; Shi et al., 2021a).

Based on this, this study collected patients with benign pulmonary nodules and lung cancer, analyzed the objective characteristics of their tongue images, and established a lung cancer classification model based on the machine learning methods. The results showed that the tongue images of lung cancer patients were dark, showing the tongue as red and crimson in color and with a coating that was thinner and more yellow than that of healthy controls and benign pulmonary nodule patients. The correlation of the objective tongue image data from patients with benign pulmonary nodules and those with lung cancer was also different, and the tongue image data-based classification model performed well in classifying benign pulmonary nodules and lung cancer.

## 2 Materials and methods

### 2.1 Participants

From July 2020 to March 2022, we collected 862 participants, including 263 patients with lung cancer at Longhua Hospital Affiliated with Shanghai University of Traditional Chinese Medicine’s Department of Oncology, 292 patients with benign pulmonary nodules, and 307 healthy subjects at Shuguang Hospital Affiliated with Shanghai University of Traditional Chinese Medicine’s Health Checkup Center. Information about tongue images was gathered, and objective tongue image data were obtained using a tongue image feature extraction system. After removing missing values and outlier samples from the tongue image data, we finally included 263 lung cancer patients, 292 benign pulmonary nodules patients, and 307 healthy controls. All lung cancer patients were diagnosed by pathology, and patients with benign pulmonary nodules were diagnosed by imaging examination or surgery. All three groups were aware of the purpose of the study and signed informed consent forms.

### 2.2 Diagnostic criteria

The diagnostic criteria for benign pulmonary nodules were small pulmonary nodules without any discomfort, in reference to the third edition of the American College of Chest Physicians’ Guidelines for the Diagnosis and Treatment of Lung Cancer (Gould et al., 2013) and “China pulmonary nodules classification, diagnosis and treatment guidelines (2016 edition)” (Zhou et al., 2016) and based on the results of imaging examination, surgery, and pathologic examination.

The lung cancer diagnostic criteria were those from the Clinical Practice Guidelines for Lung Cancer Screening issued by the National Comprehensive Cancer Network (NCCN) (Wood, 2015) and the histological classification criteria in the fourth edition of the WHO classification of lung tumors (Micke et al., 2016).

### 2.3 Inclusion and exclusion criteria

Inclusion criteria: 1) lung cancer diagnosed by pathology or cytology; 2) benign pulmonary nodules diagnosed by imaging examination, surgery, and pathological examination, with the nodules measuring less than 8 mm in size; 3) age 18–90 years; 4) complete tongue image data; and 5) understanding of the study and submission of a signed informed consent form.

Exclusion criteria were as follows: 1) inability to meet the inclusion criteria; 2) pregnancy or lactation; 3) other malignant tumors; 4) systemic acute and chronic infection; and 5) mental illness, uncooperativeness, or poor study compliance.

### 2.4 Tongue image collection

#### 2.4.1 TFDA-1 intelligent tongue diagnosis instrument

**FIGURE 1:**
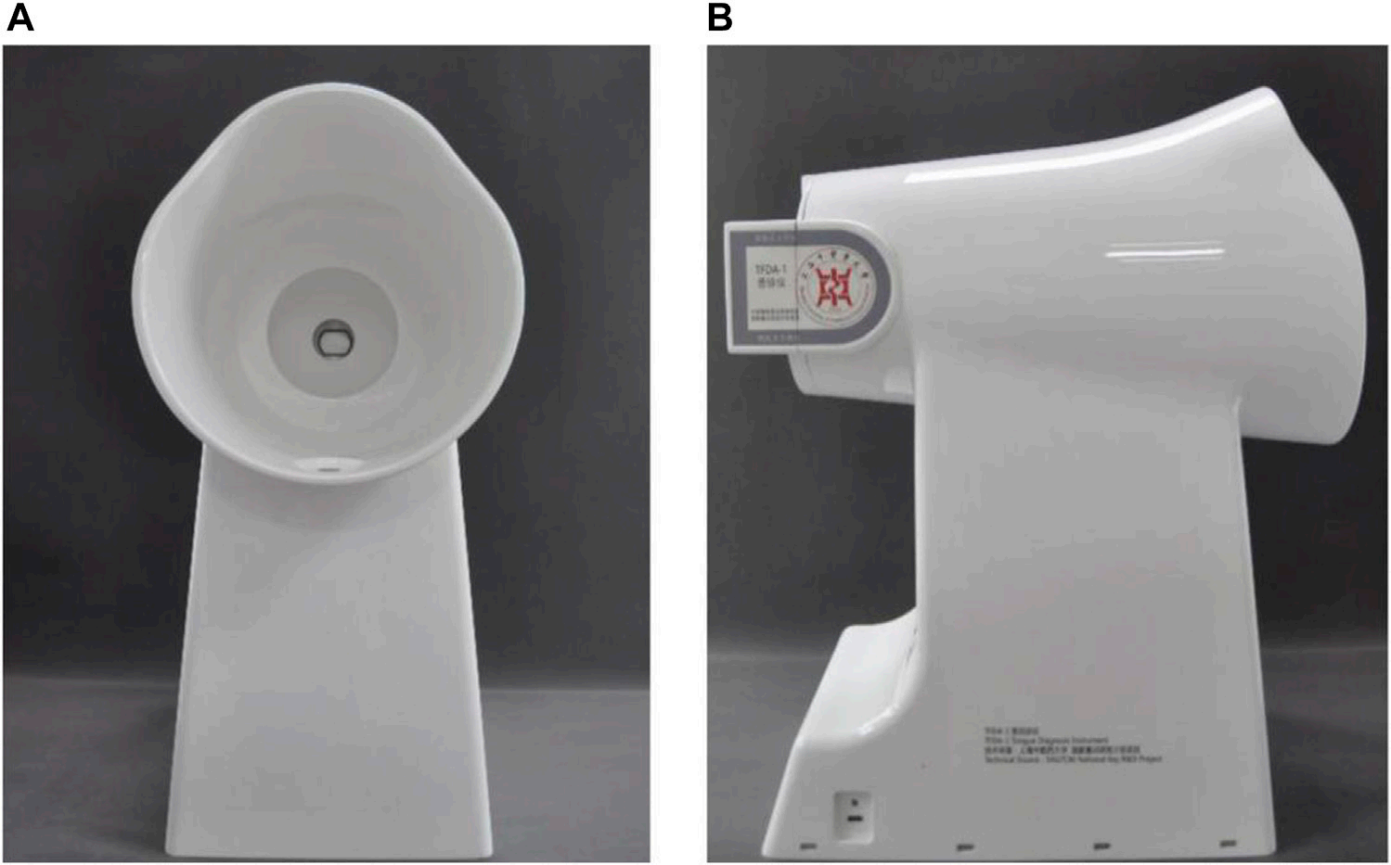
TFDA-1 digital tongue diagnosis instrument **(A)** front **(B)** side.

**FIGURE 2:**
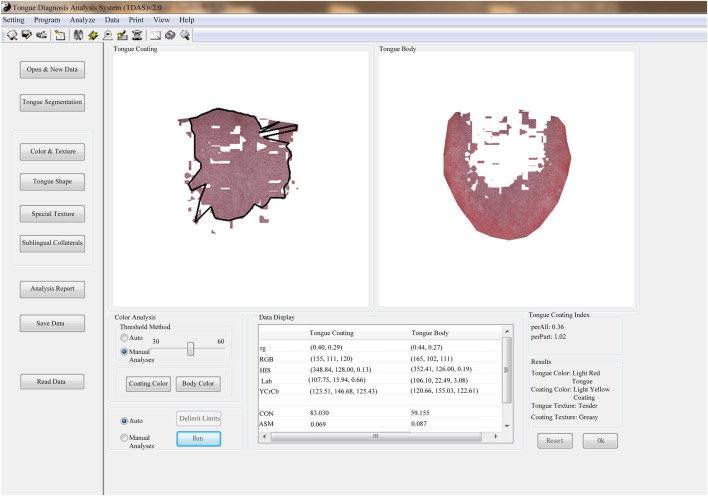
TDAS tongue image analysis system.

The tongue images of the patients were collected using the TFDA-1 digital tongue diagnosis instrument developed by the National Key Research and Development Program (NO: 2017YFC17033301) project team. The TFDA-1 digital tongue diagnosis instrument was shown in Figures 1A, B, and the corresponding tongue image analysis system TDAS was shown in Figure 2.

To ensure standardization and accuracy, all tongue images were collected by researchers who had received standardized training. The following were the specific tongue image acquisition methods: 1) set the shooting parameters and sterilize the instrument with 75% medical alcohol; 2) ask the subjects to place their chin on the jaw support of the digital tongue diagnosis instrument, relax, open their mouths and extend their tongues, relax the tongue body, flatten the tongue surface, and gently touch the center of the tongue surface to complete the acquisition; and 3) check the image taken, ensuring that the tongue surface is complete and not tense and that there is no fog cover, no light leakage, no overexposure or underexposure. Images that did not meet the aforementioned requirements were retaken.

**FIGURE 3:**
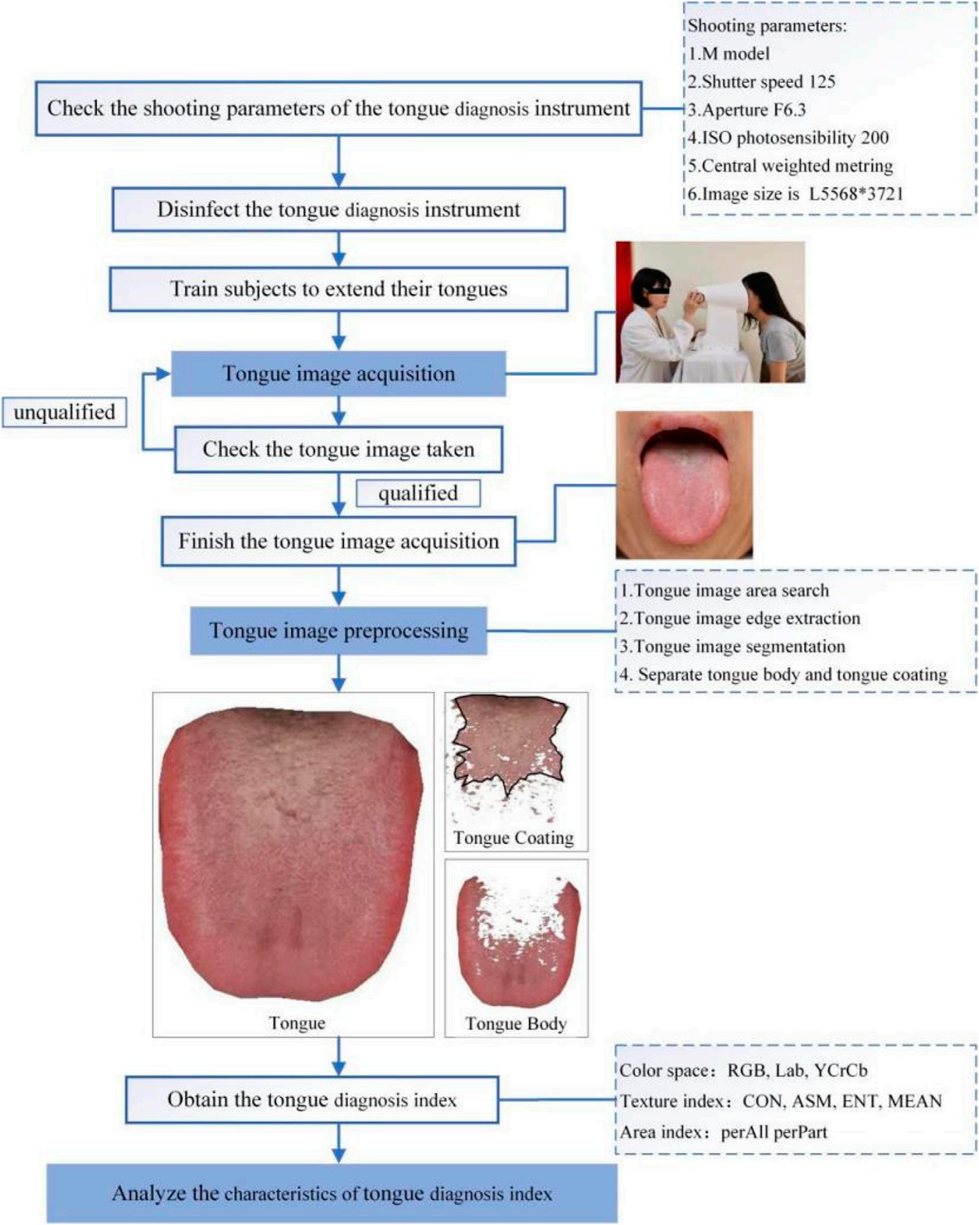
Flowchart of standardized tongue map acquisition.

The flow chart of the standardized collection and analysis of tongue images was shown in Figure 3.

#### 2.4.2 Introduction to the features of tongue images

**FIGURE 4:**
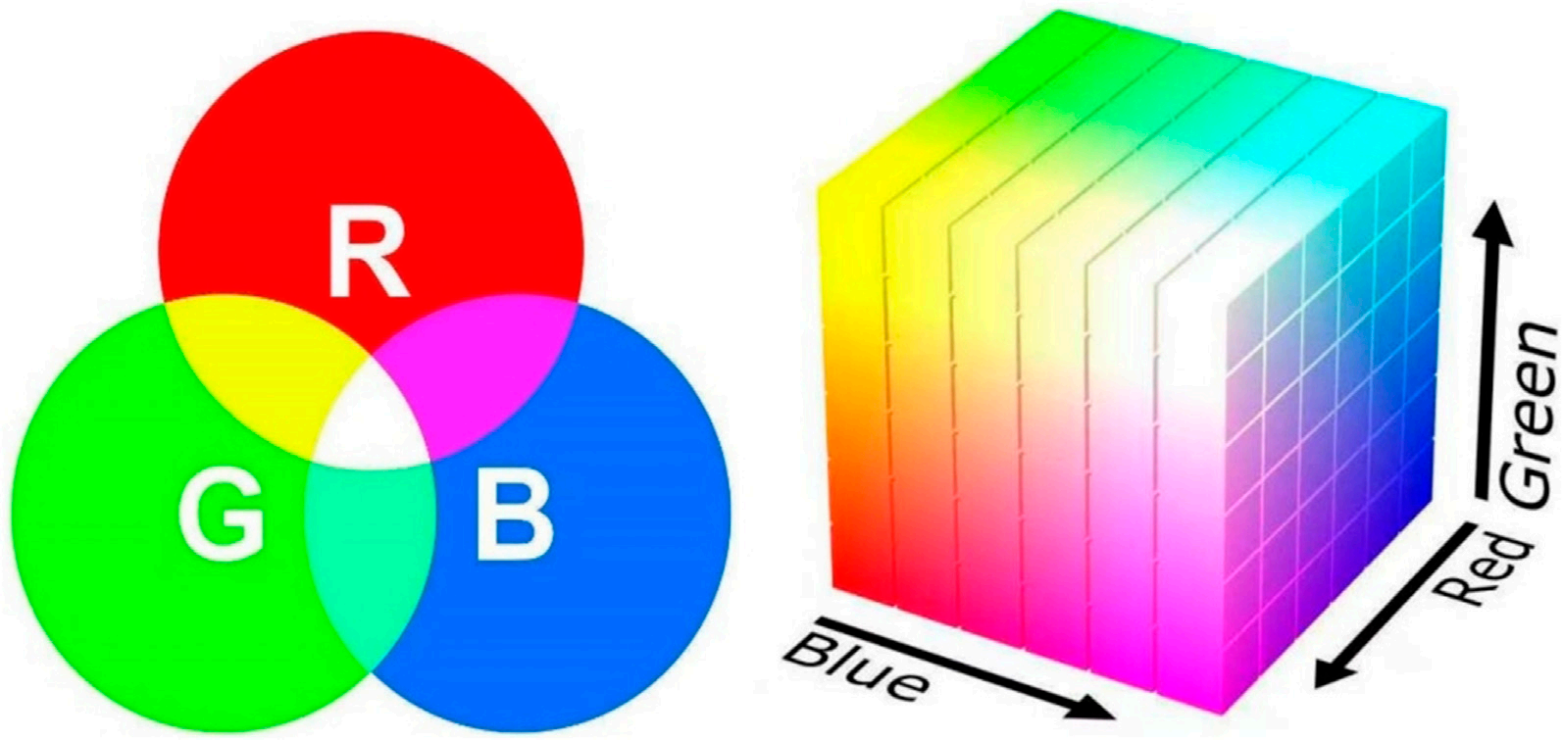
Schematic diagram of the RGB color space.

**FIGURE 5:**
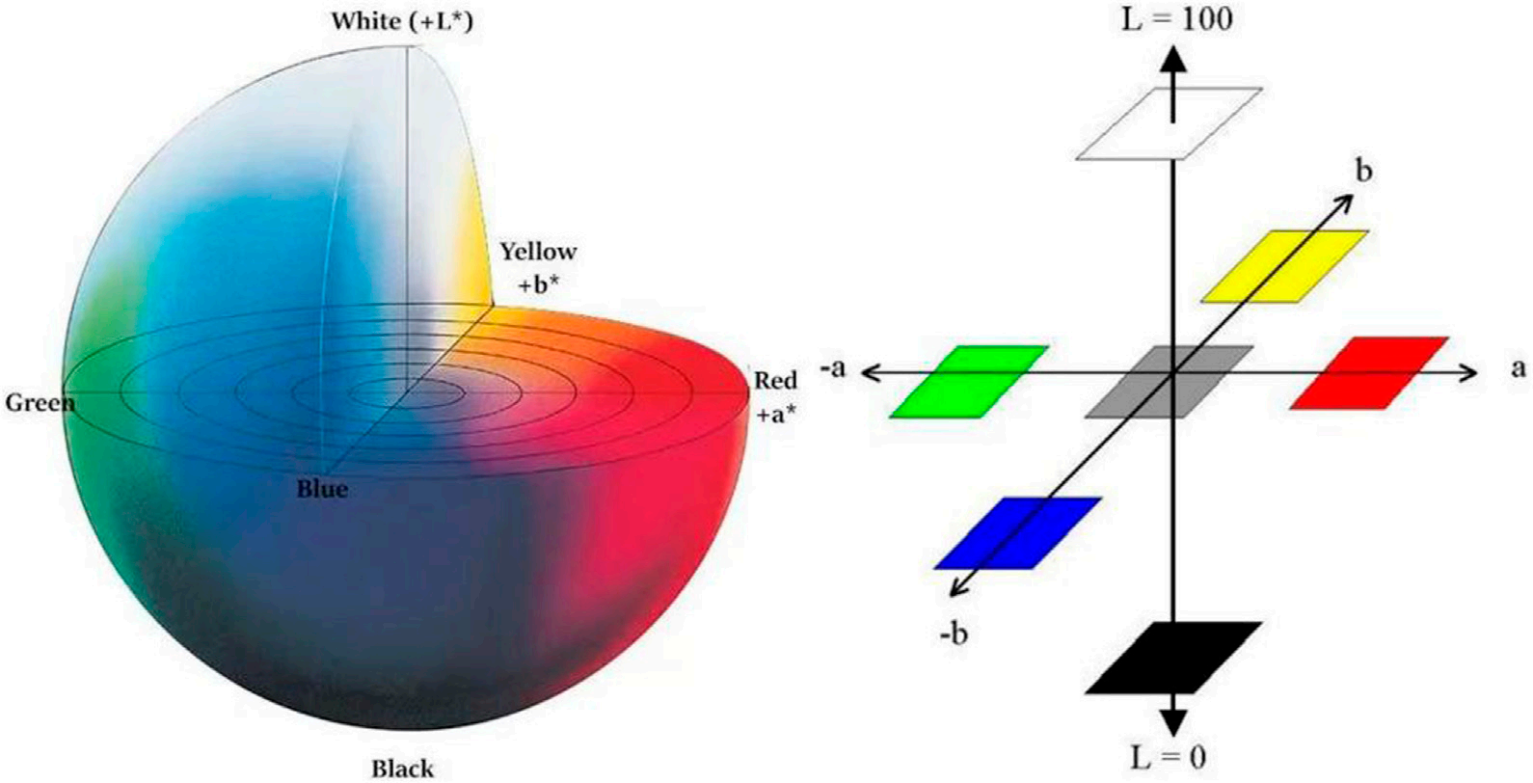
Schematic diagram of the L*a*b color space.

**FIGURE 6:**
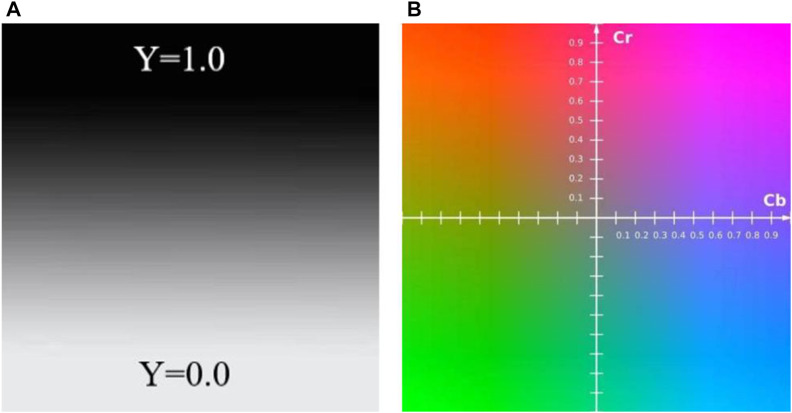
Schematic diagram of the YCrCb color space **(A)** Y color space **(B)** Cr and Cb color space.

In recent years, there are many research articles about modern tongue diagnosis technique have been published (Zhang et al., 2017; Li et al., 2021a; Jiang et al., 2021b; Li et al., 2021b; Shi et al., 2021b). In this study, we used computer technology to achieve automatic, fast and batch feature extraction of tongue images. Each tongue image could undergo repeated feature extraction, always yielding the exact same tongue image features. The tongue image color indexes were derived from RGB color space, L*a*b color space (Belasco et al., 2020), and YCrCb color space (Figures 4–6). The tongue image texture indexes include CON (Contrast), ASM (Angular Second Moment), ENT (Entropy), and MEAN. The tongue coating indexes include perAll and perPart, and the meaning of each index was shown in Table 1. In this study, the prefix “TB-” means Tongue Body, and “TC-” means Tongue Coating.

**TABLE 1:** Tongue image data and their meaning.

Tongue index	Meaning
TB/TC-R	Degree of redness of the tongue. This value ranges from 0 to 255, with larger R values indicating a redder tongue body color or a thinner tongue coating
TB/TC-G	Degree of greenness of the tongue. This value ranges from 0 to 255, with larger G values indicating a greener tongue or a paler tongue body
TB/TC-B	Degree of blueness of the tongue. This value ranges from 0 to 255, with larger B values indicating a bluer tongue or a more purple tongue body
TB/TC-Y	Luminance. This value ranges from 16 to 235, with smaller Y values indicating a darker tongue
TB/TC-Cr	Chrominance. This value ranges from 16 to 240 and reflects the difference between the red part of the RGB input signal and the brightness value of the RGB signal
TB/TC-Cb	Chrominance. This value ranges from 16 to 240 and reflects the difference between the blue part of RGB input signal and the brightness value of RGB signal
TB/TC-L	Lightness. This value ranges from 0 to 100 represents the range from pure black to pure white
TB/TC-a	The green‒red axis. A positive value represents red, and a negative value represents green
TB/TC-b	The blue‒yellow axis. A positive value represents yellow, and a negative value represents blue
TB/TC-CON Jordanova et al. (2022)	The clarity of the image and the depth of the texture groove
TB/TC-ENT Siqueira et al. (2009), Küçükaslan et al. (2014)	The degree of nonuniformity or complexity of the tongue texture
TB/TC-ASM Jordanova et al. (2022)	The tongue grayscale distribution uniformity and texture thickness
TB/TC-MEAN Zabitler et al. (2022)	Grayscale mean of the tongue
perAll	The ratio of the pixels representing the tongue coating to the total tongue area
perPart	The ratio of the pixels representing the tongue coating to the area occupied by the tongue coating

### 2.5 Statistical analysis

SPSS 26.0 was used for statistical analysis. Counting data were expressed as frequencies and constituent ratios, and the chi-square test was used for comparisons between groups. For continuous measurement data, the normality test was performed. Tongue image data that conformed to a normal distribution were expressed as the mean and standard deviation (SD), while tongue image data that did not conform to a normal distribution were expressed as the median and quartile. Measurement data that conformed to a normal distribution and demonstrated homogeneity of variance were compared among multiple groups using ANOVA, and measurement data that did not conform to a normal distribution and homogeneity of variance were compared using the Kruskal‒Wallis H rank-sum test. For bivariate correlation analysis, Pearson’s correlation was used for normally distributed variables, and Spearman’s correlation was used for nonnormally distributed variables. All tests were two-tailed, and *p* < 0.05 was considered statistically significant.

### 2.6 Modeling

We used six machine learning algorithms, including decision tree, SVM, random forest, neural network, naïve Bayes, and logistic regression, to build prediction models of lung cancer. Logistic regression is a popular supervised machine learning technique that creates prediction models by determining the relationship between independent and dependent variables, and it is primarily used to solve classification problems (Schober and Vetter, 2021a). Its derivation process and calculation method are similar to regression, and it is considered an extension of linear regression (Schober and Vetter, 2021b). According to studies, logistic regression does not demonstrate a worse classification ability than other machine learning methods (Christodoulou et al., 2019; Song et al., 2021). Logistic regression analysis can be used to estimate the probability of a certain output class based on some input variables (Meurer and Tolles, 2017; Xiang et al., 2022). Decision tree is a classical machine learning algorithm that can be used for classification and regression problems. It can be used to divide data step by step according to the training data and partition the data according to feature attributes to achieve classification or make predictions (Pashaei et al., 2015; Wu et al., 2020). Bayesian classification is a machine learning method based on Bayes’ theorem, which classifies samples by calculating the probability that a sample belongs to a certain class (Asafu-Adjei and Betensky, 2015; Ramanujam and Kaliappan, 2016). Support vector machine is a kind of classification and regression algorithm that can project data into a high-dimensional space; by finding the optimal segmentation plane in the high-dimensional space, the data can be classified or regressed (Agyapong et al., 2022; Tang et al., 2022). Neural networks are artificial networks that mimic the workings of neural networks in the human brain and can be used to solve classification, regression, and a variety of other machine learning problems (Checcucci et al., 2020; Laudicella et al., 2021). The random forest algorithm is an integrated learning algorithm based on a decision tree, which is a statistical extension of the classification and regression tree (CART) algorithm. It constructs multiple decision trees and then combines them together. An important concept in random forest is randomness, which provides a good method for reducing overfitting (Chen et al., 2014; Seo et al., 2019).

In this study, we performed the modeling using R language program modeling. Ten-fold cross-validation was used to screen the best parameters for the models. After confirming the optimal parameters, they were locked, and the data were divided into a training set and a test set at a ratio of 7:3. To avoid the contingency of one modeling’s results, we performed three random samplings and used the means and standard deviations of the three modeling results to evaluate the model’s classification efficiency. Accuracy, sensitivity, specificity, F1 score, precision, area under the curve (AUC), positive predictive value (PPV), negative predictive value (NPV), and area under the precision-recall curve (AUC_PR_) which is average precision (AP) were used to assess the model’s performance. Formulas of The formulas for accuracy, sensitivity, specificity, precision, and F1 score were as follows:

$$Accuracy=\frac{TP+TN}{TP+TN+FP+FN}$$ (1)

$$Sensitivity=\frac{TP}{TP+FN}$$ (2)

$$Specificity=\frac{TN}{TN+FP}$$ (3)

$$Precision=\frac{TP}{TP+FP}$$ (4)

$$F1=\frac{2\times Precision\times Sensitivity}{Precision+Sensitivity}$$ (5)

$$PPV=\frac{TP}{TP+FP}$$ (6)

$$NPV=\frac{TN}{TN+FN}$$ (7)

## 3 Results

### 3.1 Characteristics of the participants

The baseline characteristics of the three groups were shown in Table 2.

**TABLE 2:** Baseline characteristics of the study participants.

/		Healthy controls (*n* = 307)	Benign pulmonary nodules (*n* = 292)	Lung cancer (*n* = 263)
Sex [*n* (%)]	Male	143 (46.58)	148 (50.68)	124 (47.15)
	Female	164 (53.42)	144 (49.32)	139 (52.85)
Age [Mean (SD)]		36.30 ± 6.34	42.91 ± 10.89**	57.05 ± 15.21**^△△^

Compared with healthy controls, *p < 0.05, **p < 0.01. Compared with benign pulmonary nodules, ^△^ *p* < 0.05, ^△△^ *p* < 0.01.

The results revealed that there was no statistically significant difference in sex distribution among the three groups (*p* > 0.05), but there were statistically significant differences in age (*p* < 0.01). Patients’ ages in the lung cancer group was significantly older than the benign pulmonary nodules group, and the group with benign pulmonary nodules was significantly older than the healthy controls. Because no study has demonstrated that age has a significant effect on the characteristic of the tongue, no age matching was performed among the participants in this study.

### 3.2 Statistical analysis of tongue image data

The statistical analysis results for the tongue image data of the three groups were shown in Table 3.

**TABLE 3:** Comparison results of tongue image data of the three groups [Mean (SD), Median (P_25_, P_75_)].

Tongue index			Healthy controls (*n* = 307)	Benign pulmonary nodules (*n* = 292)	Lung cancer (*n* = 263)
TB	RGB	R	162.00 (158.00, 166.000)	161.00 (157.00, 164.00)**	160.00 (156.00, 164.00)**
		G	97.00 (92.00, 102.00)	97.00 (93.00, 102.00)	93.00 (88.00, 98.00)**^△△^
		B	101.00 (97.00, 109.00)	102.00 (96.00, 106.00)	96.00 (90.00, 101.00)**^△△^
	Lab	L	48.40 (47.02, 50.42)	48.37 (46.92, 49.81)	47.26 (45.62, 48.78)**^△△^
		a	26.80 ± 2.15	26.28 ± 2.43*	27.75 ± 2.30**^△△^
		b	8.82 ± 1.57	8.69 ± 1.86	10.38 ± 2.27**^△△^
	YCrCb	Y	116.19 (113.20, 120.68)	116.30 (113.08,119.43)	113.60 (109.73, 117.11)**^△△^
		Cr	156.14 ± 1.94	155.59 ± 2.29*	157.30 ± 2.21**^△△^
		Cb	120.31 ± 1.19	120.41 ± 1.39	119.26 ± 1.69**^△△^
	Texture index	CON	103.20 (84.93, 128.01)	105.54 (86.67, 123.55)	96.17 (83.35, 113.41)*^△^
		ASM	0.06 (0.05, 0.07)	0.06 (0.06, 0.07)	0.06 (0.06, 0.07)*^△^
		ENT	1.29 (1.25, 1.34)	1.30 (1.25, 1.33)	1.28 (1.24, 1.32)*
		MEAN	0.03 (0.03, 0.04)	0.03 (0.03, 0.03)	0.03 (0.03,0.03)**^△^
TC	RGB	R	151.00 (144.00, 158.00)	152.00 (145.00, 158.00)	148.00 (136.00, 156.00)**^△△^
		G	105.00 (98.00, 111.00)	106.00 (99.25, 113.00)	99.00 (88.00, 107.00)**^△△^
		B	107.00 (100.00, 114.00)	108.00 (100.00, 115.00)	101.00 (87.00, 108.00)**^△△^
	Lab	L	49.17 (46.47, 51.79)	49.54 (47.08, 51.95)	47.21 (42.35, 50.20)**^△△^
		a	19.01 ± 1.90	18.46 ± 2.31**	19.66 ± 2.03**^△△^
		b	6.03 (5.02, 7.19)	6.08 (4.97, 7.45)	7.59 (6.09, 9.29)**^△△^
	YCrCb	Y	118.20 (112.14, 123.08)	118.95 (113.50, 124.25)	113.86 (102.96, 120.41)**^△△^
		Cr	148.21 ± 1.72	147.74 ± 2.10*	149.02 ± 1.89**^△△^
		Cb	122.21 (121.47, 123.08)	122.21 (121.19, 123.09)	121.19 (120.00, 122.35)**^△△^
	Texture index	CON	129.86 (104.92, 157.78)	135.67 (108.38, 167.48)	130.33 (99.64, 155.93)
		ASM	0.05 (0.05, 0.06)	0.05 (0.05, 0.06)	0.06 (0.05, 0.06)^△△^
		ENT	1.34 (1.29, 1.39)	1.35 (1.30, 1.40)	1.34 (1.27, 1.38)^△△^
		MEAN	0.04 (0.03, 0.04)	0.04 (0.03, 0.04)	0.04 (0.03, 0.04)
	Area index	perAll	0.33 (0.27, 0.38)	0.34 (0.28, 0.41)	0.23 (0.11, 0.33)**^△△^
		perPart	0.65 (0.57, 0.76)	0.69 (0.58, 0.87)*	0.64 (0.53, 0.83)^△△^

Compared with healthy controls, *p < 0.05, **p < 0.01. Compared with benign pulmonary nodules, ^△^ *p* < 0.05, ^△△^ *p* < 0.01.

**FIGURE 7:**
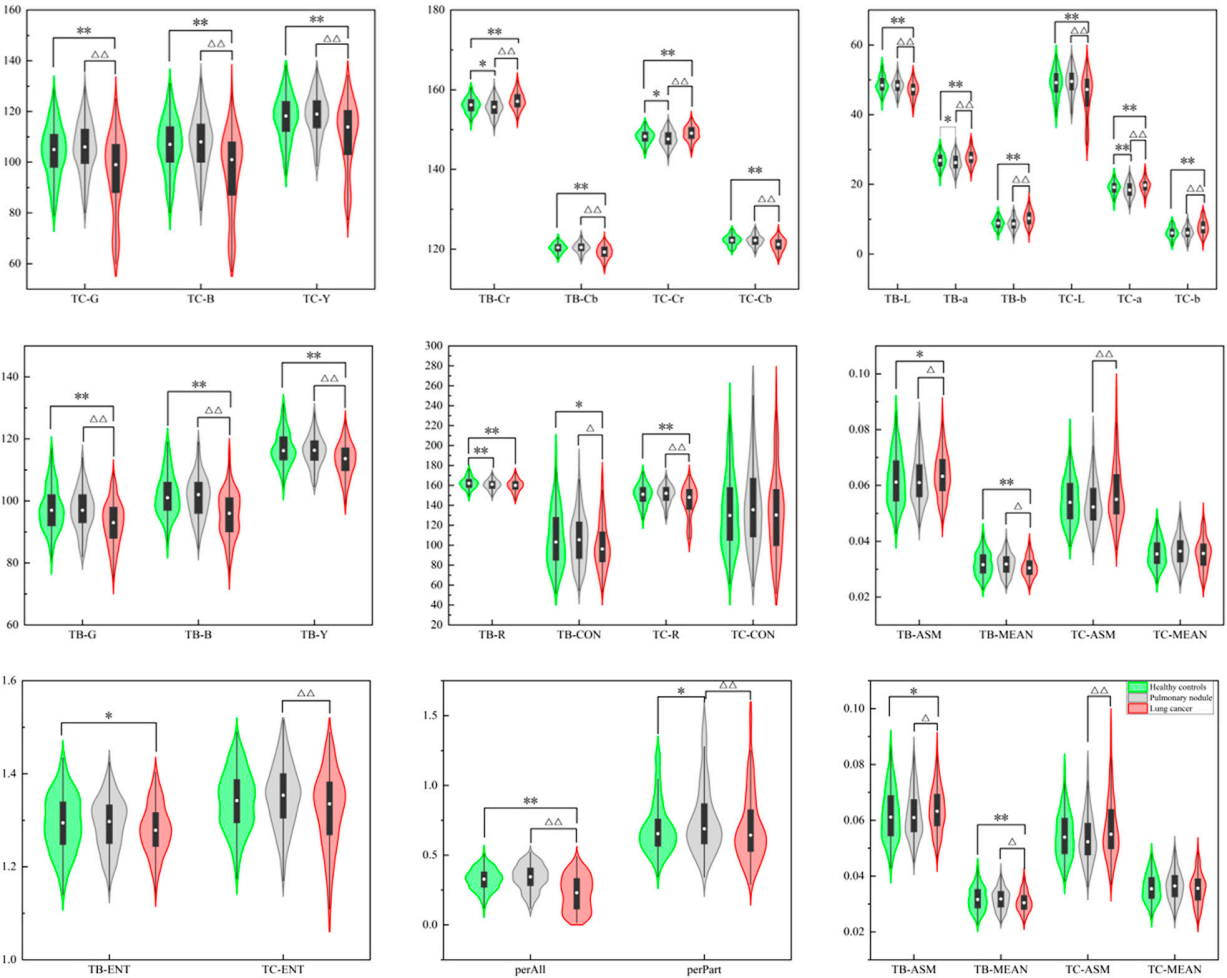
Violin map of tongue image feature distribution of the three groups.

The violin maps shown in Figure 7 were created using the Origin 2021 software to facilitate observation of the distribution of tongue image indexes.

The results showed that there were significant differences in the L, a, b, Y, Cr, and Cb values of the tongue body and coating among the three groups (*p* < 0.01). The lung cancer group’s TB-L, TC-L, TB-Y, and TC-Y values were lower than those of the benign pulmonary nodules group (*p* < 0.01). The values of TB-a, TC-a, TB-Cr, and TC-Cr of the lung cancer group were significantly higher than those of the benign pulmonary nodules group (*p* < 0.01), while TB-B, TC-B, TB-Cb, and TC-Cb were significantly lower (*p* < 0.01). The TB-b and TC-b values of the lung cancer group were significantly higher than those of the benign pulmonary nodules group (*p* < 0.01). The TC-L, TB-Y, TC-Y, TB-G, TC-G, TB-B, TC-B, TB-Cb, and TC-Cb values of the benign pulmonary nodules group were higher than those of the healthy controls (*p* < 0.01), while the TB-a, TC-a, TB-Cr, TC-Cr values were lower (*p* < 0.01). The value of perAll in the lung cancer group was lower than that in the benign pulmonary nodules group (*p* < 0.01), and in the benign pulmonary nodules group, it was larger than that in the healthy controls (*p* < 0.01). Furthermore, the TC-ASM was higher in the lung cancer group than in the benign pulmonary nodules group, but the other texture indexes (CON, ENT, MEAN) were not significantly different between the two groups.

### 3.3 Correlation analysis of tongue image data

**FIGURE 8:**
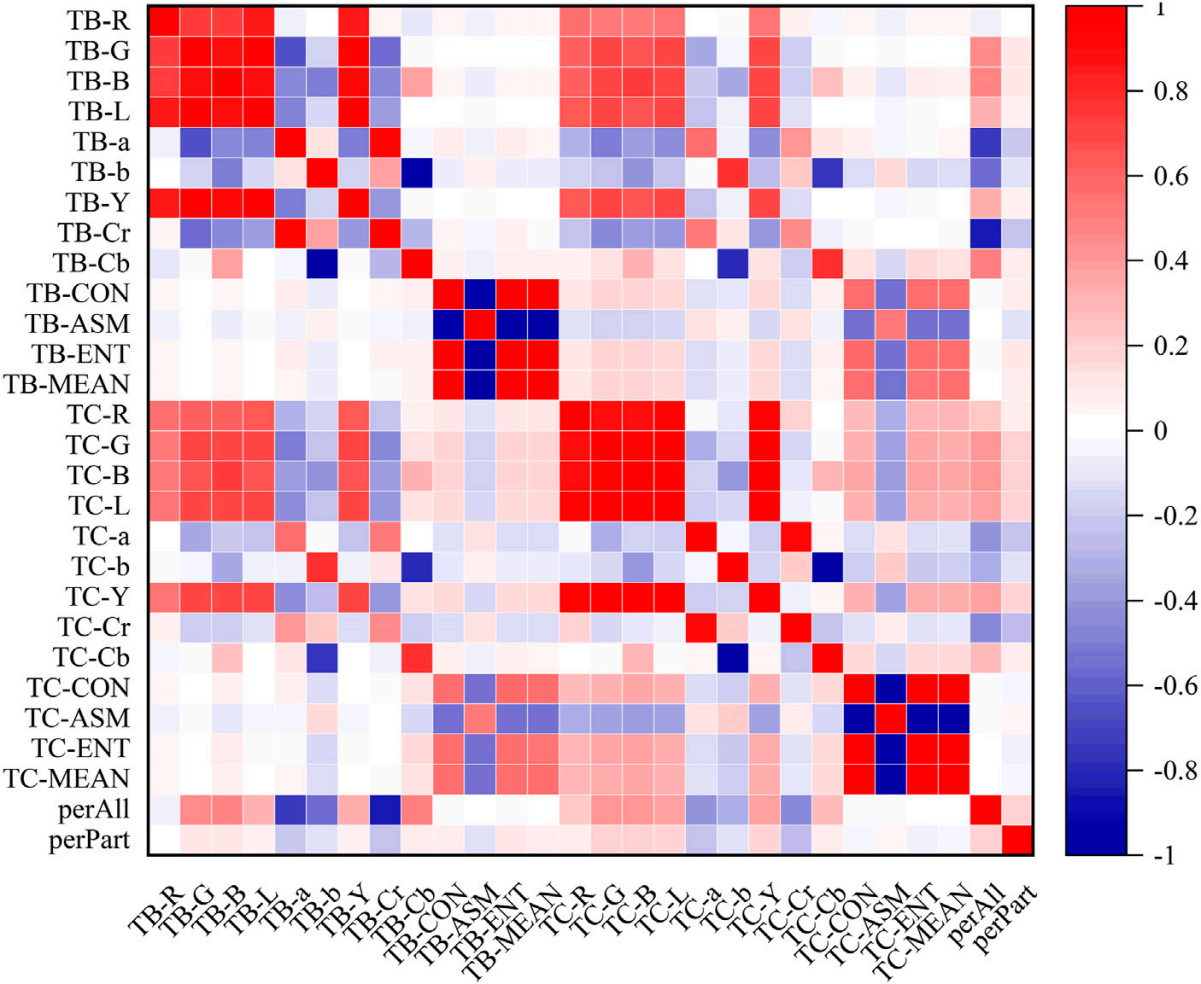
Correlation heatmap of the healthy control group.

**FIGURE 9:**
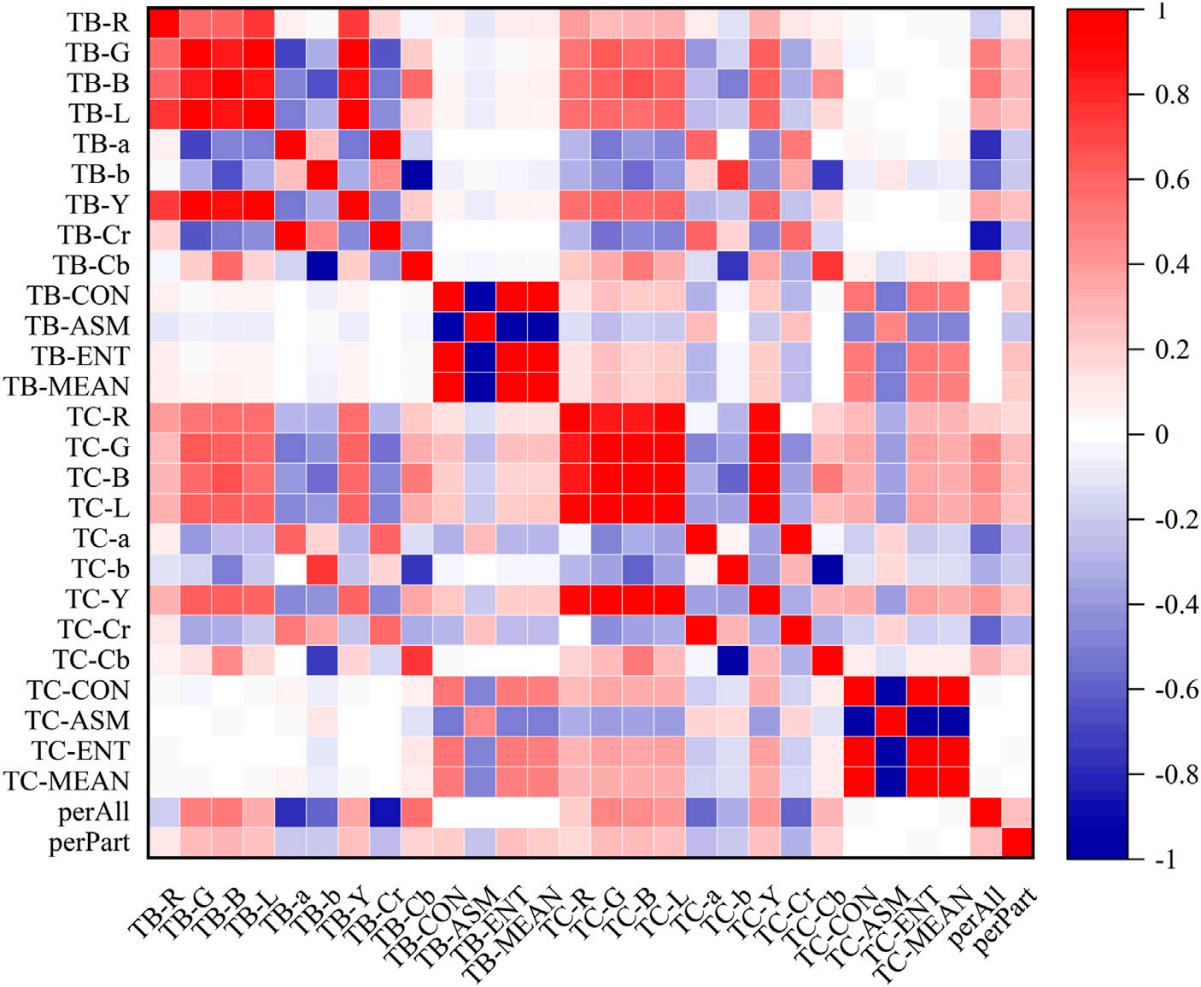
Correlation heatmap of the benign pulmonary nodules group.

**FIGURE 10:**
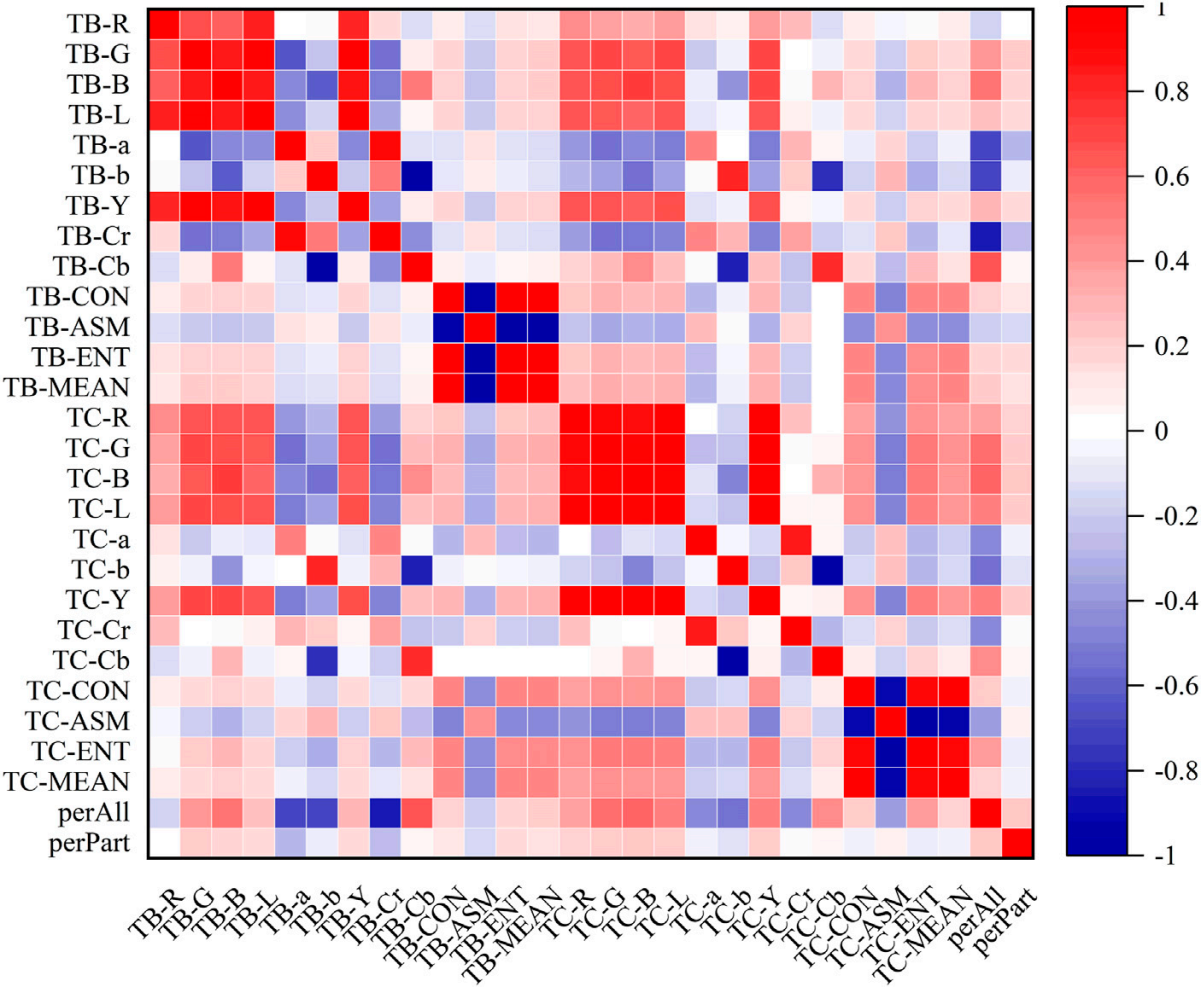
Correlation heatmap of the lung cancer group.

In the heatmaps, the red square represents a positive correlation, and the blue square represents a negative correlation. The heatmaps of the correlation analysis results of the tongue indexes in the healthy control group, benign pulmonary nodules group, and lung cancer group were shown in Figures 8–10, and the corresponding correlation coefficient values of the tongue image data for the three groups were shown Supplementary Figures S1–S3.

According to the results, the correlations between the texture indexes of the tongue body and tongue coating (TB/TC-CON, TB/TC-ASM, TB/TC-ENT, TB/TC-MEAN) and the RGB\Lab\YCrCb color space indexes of the tongue body showed the following trends: lung cancer group > healthy controls > benign pulmonary nodules group. The correlation between TC-a and TB-G and between TC-Cr and TB-G, TB-B, and TB-L, showed the following trends: benign pulmonary nodules group > healthy controls > lung cancer group. In the group of people with lung cancer, the correlation coefficient of TB-Cr and TB-a was 0.93 (*p* ≤ 0.001), while in the group of people with benign pulmonary nodules, it was 0.96 (*p* ≤ 0.001). The correlation coefficients between TC-b and TB-b and TB-Cb in the group of people with lung cancer were 0.83 and −0.85, respectively (*p* ≤ 0.001), while those between TC-Cb and TB-b and TB-Cb were −0.78 and 0.82 (*p* ≤ 0.001). In contrast, in the group of people with benign pulmonary nodules, the correlations of TC-b with TB-b and TB-Cb were 0.77 and −0.75 (*p* ≤ 0.001), and those of TC-Cb with TB-b and TB-Cb were −0.75 and 0.77 (*p* ≤ 0.001), respectively. However, the correlations between the texture parameters of the tongue body and tongue coating in the benign pulmonary nodules group were higher than those in the lung cancer group.

**FIGURE 11:**
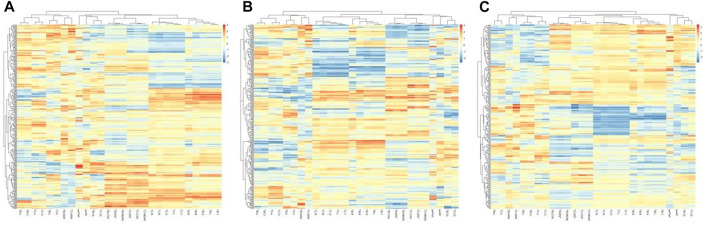
Hierarchical clustering of tongue indexes of the three groups. **(A)** the healthy controls, **(B)** the benign pulmonary nodules group, **(C)** the lung cancer group.

The hierarchical clustering heatmaps of the three groups of tongue indexes were shown in Figure 11.

The results showed that the healthy control group and benign pulmonary nodules group had similar clusterings, while the clustering results of the lung cancer group were significantly different from those of the other two groups.

### 3.4 Modeling results

First, collinearity statistics were calculated for the tongue image data that were significantly different between benign pulmonary nodules and lung cancer patients. Collinearity was considered present if the tolerance was less than 0.1 or the variance inflation factor (VIF) was larger than 10. The following indexes were assessed and found to be noncollinear after factors that could have led to collinearity in this study were eliminated. The statistics on collinearity were shown in Table 4.

**TABLE 4:** Collinearity statistics of tongue image data.

Index	Tolerance	VIF	Index	Tolerance	VIF
TB-L	0.415	2.412	TC-b	0.246	4.067
TB-a	0.202	4.952	perAll	0.124	8.066
TB-b	0.179	5.574	perPart	0.809	1.236
TC-L	0.302	3.307	TB-ASM	0.641	1.56
TC-a	0.502	1.992	TC-ASM	0.489	2.045

This study mainly to analyze the contribution rate of tongue image data to the differential diagnosis of lung cancer and benign pulmonary nodules. Meanwhile, the baseline information, baseline information combined with tongue image data were respectively used as input variables for modeling to construct different models, which in order to compare the modeling effect of tongue image data. The model evaluation results of the six machine learning methods were shown in Table 5.

**TABLE 5:** Classification results of each model based on different data sets [mean (standard deviations)].

Classifier	Data set	Sensitivity	Specificity	F1_score	Precision	Accuracy	AUC	PPV	NPV	AP
Decision tree	Model 1	0.549 (0.030)	0.790 (0.057)	0.606 (0.031)	0.676 (0.044)	0.689 (0.032)	0.717 (0.007)	0.835 (0.062)	0.735 (0.019)	0.776 (0.026)
	Model 2	0.491 (0.045)	0.748 (0.076)	0.545 (0.026)	0.628 (0.080)	0.631 (0.020)	0.679 (0.022)	0.596 (0.029)	0.702 (0.029)	0.635 (0.042)
	Model 3	0.589 (0.072)	0.911 (0.007)	0.692 (0.054)	0.846 (0.019)	0.764 (0.043)	0.764 (0.033)	0.709 (0.089)	0.865 (0.008)	0.741 (0.022)
SVM	Model 1	0.650 (0.029)	0.768 (0.027)	0.664 (0.027)	0.683 (0.063)	0.715 (0.012)	0.703 (0.008)	0.745 (0.012)	0.867 (0.011)	0.806 (0.007)
	Model 2	0.633 (0.058)	0.780 (0.030)	0.665 (0.021)	0.706 (0.034)	0.711 (0.033)	0.694 (0.033)	0.655 (0.012)	0.721 (0.042)	0.759 (0.026)
	Model 3	0.687 (0.048)	0.851 (0.014)	0.734 (0.034)	0.791 (0.043)	0.774 (0.029)	0.755 (0.027)	0.76 (0.048)	0.824 (0.014)	0.844 (0.022)
Random forest	Model 1	0.560 (0.021)	0.766 (0.014)	0.600 (0.030)	0.648 (0.051)	0.677 (0.005)	0.697 (0.01)	0.768 (0.013)	0.779 (0.025)	0.623 (0.038)
	Model 2	0.586 (0.066)	0.761 (0.044)	0.623 (0.038)	0.673 (0.047)	0.679 (0.048)	0.752 (0.051)	0.628 (0.031)	0.660 (0.011)	0.741 (0.025)
	Model 3	0.660 (0.047)	0.862 (0.041)	0.724 (0.045)	0.802 (0.042)	0.770 (0.050)	0.804 (0.029)	0.732 (0.052)	0.778 (0.026)	0.841 (0.027)
Neural network	Model 1	0.626 (0.017)	0.829 (0.014)	0.677 (0.022)	0.738 (0.037)	0.741 (0.020)	0.749 (0.010)	0.746 (0.016)	0.689 (0.036)	0.806 (0.006)
	Model 2	0.585 (0.081)	0.744 (0.087)	0.614 (0.018)	0.666 (0.073)	0.669 (0.037)	0.690 (0.058)	0.631 (0.051)	0.664 (0.036)	0.676 (0.077)
	Model 3	0.612 (0.103)	0.897 (0.040)	0.697 (0.078)	0.827 (0.073)	0.762 (0.059)	0.777 (0.044)	0.712 (0.063)	0.895 (0.025)	0.811 (0.007)
Naive bayes	Model 1	0.637 (0.020)	0.797 (0.039)	0.668 (0.029)	0.707 (0.070)	0.725 (0.022)	0.760 (0.011)	0.753 (0.021)	0.912 (0.026)	0.805 (0.008)
	Model 2	0.642 (0.071)	0.690 (0.031)	0.636 (0.044)	0.633 (0.027)	0.667 (0.052)	0.748 (0.047)	0.649 (0.027)	0.662 (0.015)	0.712 (0.057)
	Model 3	0.672 (0.068)	0.741 (0.033)	0.677 (0.045)	0.684 (0.024)	0.709 (0.052)	0.795 (0.039)	0.67 (0.065)	0.697 (0.040)	0.806 (0.034)
Logistic regression	Model 1	0.697 (0.034)	0.723 (0.033)	0.675 (0.019)	0.659 (0.062)	0.709 (0.006)	0.758 (0.010)	0.790 (0.015)	0.823 (0.009)	0.806 (0.006)
	Model 2	0.662 (0.041)	0.713 (0.044)	0.659 (0.022)	0.659 (0.037)	0.689 (0.038)	0.745 (0.041)	0.661 (0.016)	0.678 (0.030)	0.758 (0.028)
	Model 3	0.744 (0.036)	0.776 (0.006)	0.738 (0.019)	0.734 (0.028)	0.760 (0.021)	0.808 (0.031)	0.801 (0.053)	0.750 (0.007)	0.857 (0.031)

Note: Model 1, model based on baseline data (age and sex), Model 2, model based on tongue image data, Model 3, model based on both baseline and tongue image data, PPV, positive predictive value; NPV, negative predictive value; AP, average precis.

**FIGURE 12:**
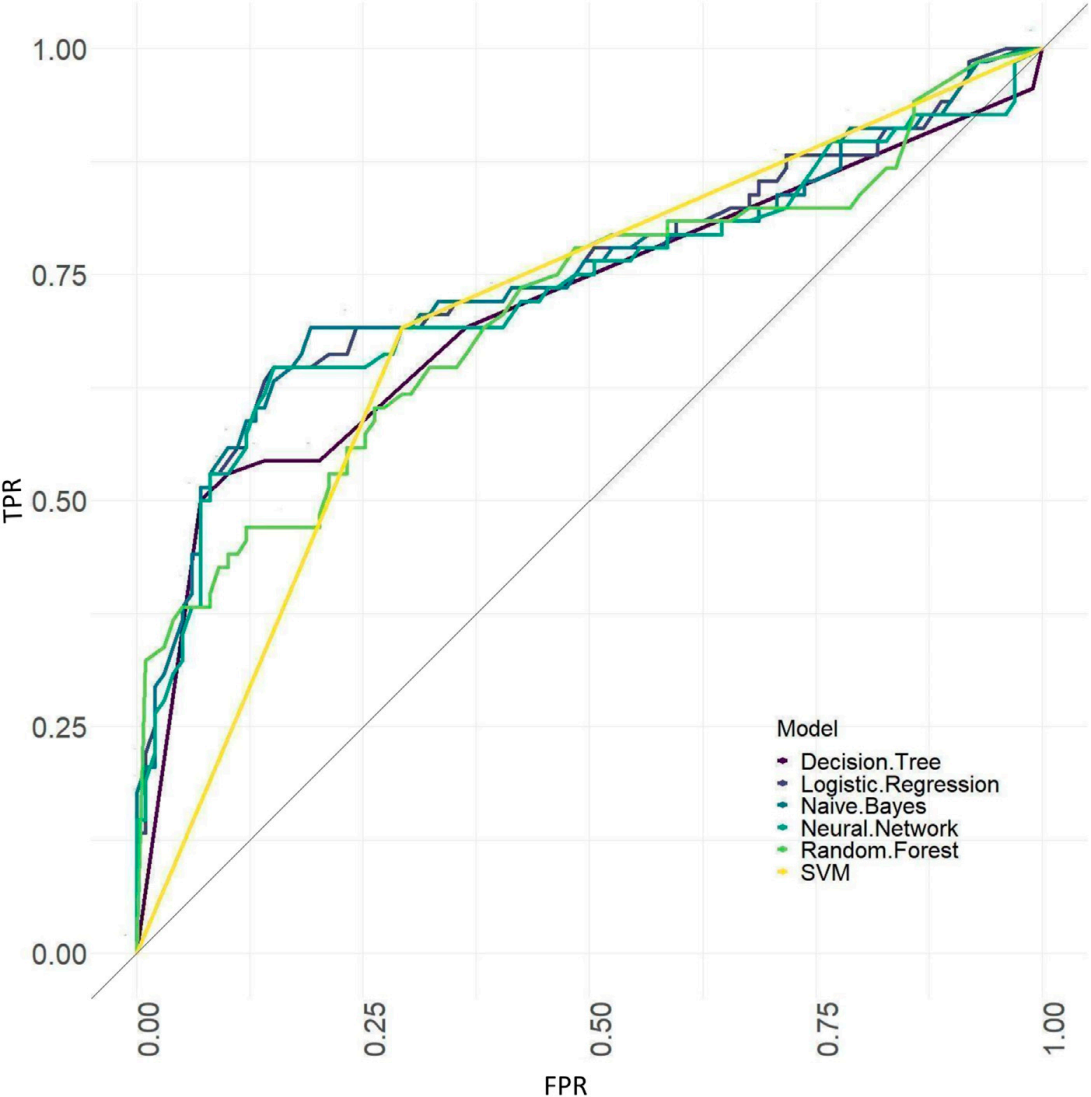
ROC curves of the models based on baseline data sets.

**FIGURE 13:**
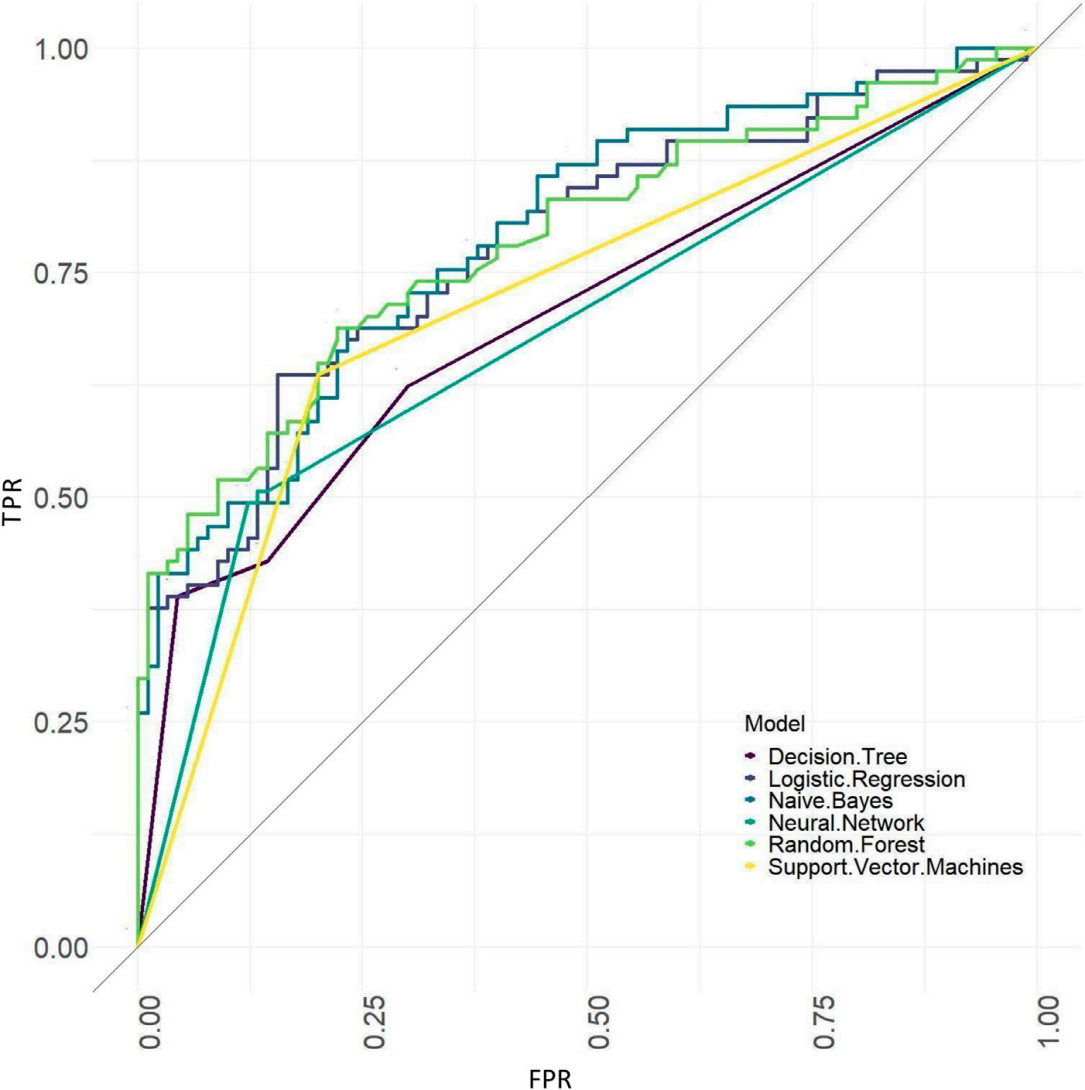
ROC curves of the models based on tongue image data.

**FIGURE 14:**
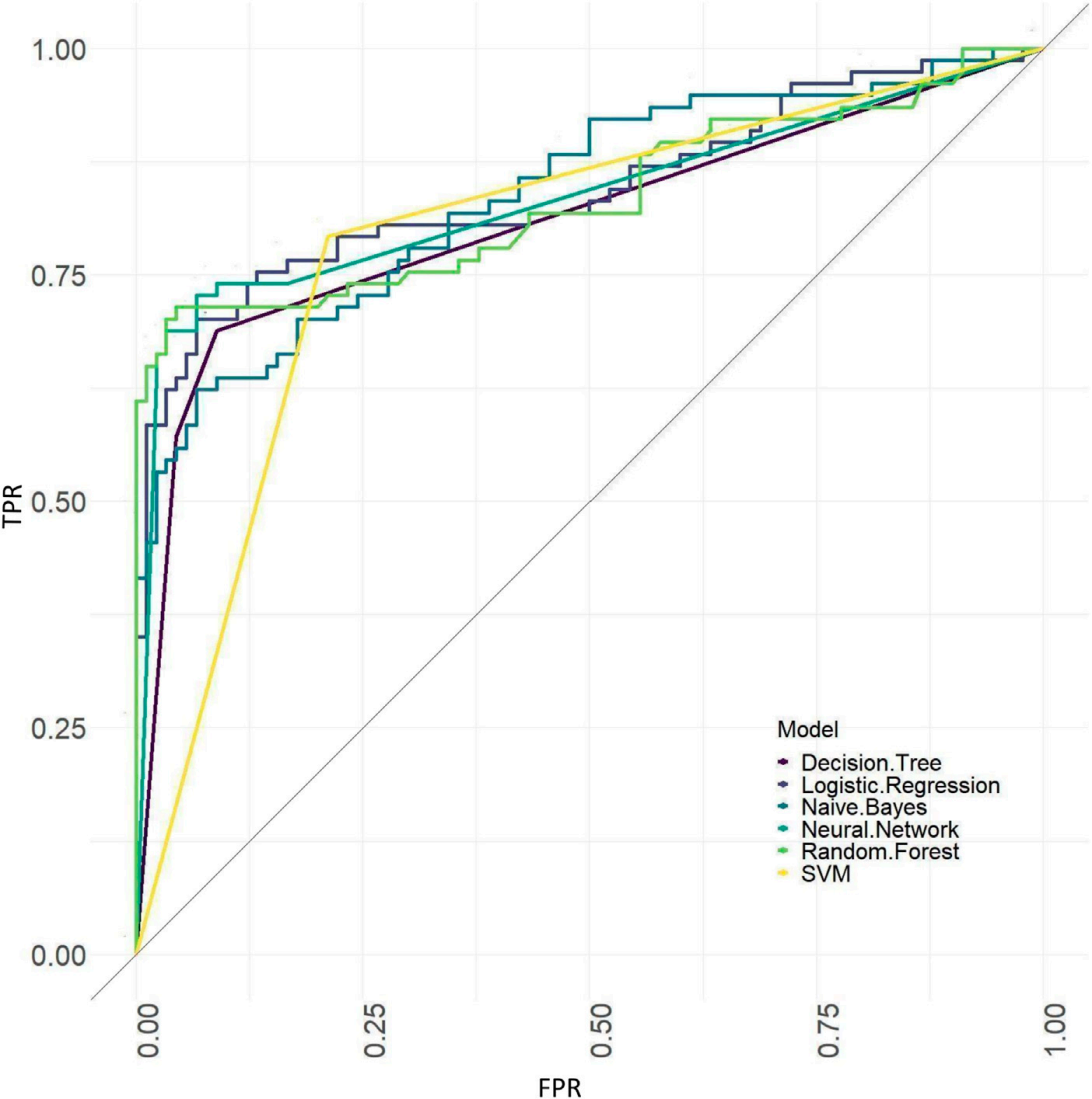
ROC curves of the models based on baseline and tongue image data.

The ROC curves of models based on baseline, tongue image data, baseline and tongue image data using each machine learning algorithm were shown in Figures 12–14.

The precision-recall curves of models based on baseline, tongue image data, baseline and tongue image data were shown in Supplementary Figures S4–S6. The precision-recall curves from A to F in Supplementary Figures S4–S6 correspond to various algorithms such as decision tree, SVM, random forest, neural network, naive bayes, and logistic regression, respectively.

The results showed that of the models based on tongue image data, the random forest prediction model performed the best, with a model accuracy of 0.679 ± 0.048 and an AUC of 0.752 ± 0.051, while among models based on the baseline data, the naïve Bayes prediction model performed the best, with a model accuracy of 0.725 ± 0.022 and an AUC of 0.760 ± 0.011. In addition, all models based on baseline data (except the random forest model) outperformed the tongue data-based models. There was no significant difference in the model performance of multiple machine learning methods in the “baseline and tongue image” data set. The accuracy for the logistic regression, decision tree, support vector machine, random forest, neural network, and naïve bayes models based on both the baseline and tongue image data were 0.760 ± 0.021, 0.764 ± 0.043, 0.774 ± 0.029, 0.770 ± 0.050, 0.762 ± 0.059, and 0.709 ± 0.052, respectively, while the AUCs for each model were 0.808 ± 0.031, 0.764 ± 0.033, 0.755 ± 0.027, 0.804 ± 0.029, 0.777 ± 0.044, and 0.795 ± 0.039, respectively. Furthermore, all six machine learning methods performed better when based on both the baseline and tongue image data than when based only on the tongue image data or on the baseline data.

**FIGURE 15:**
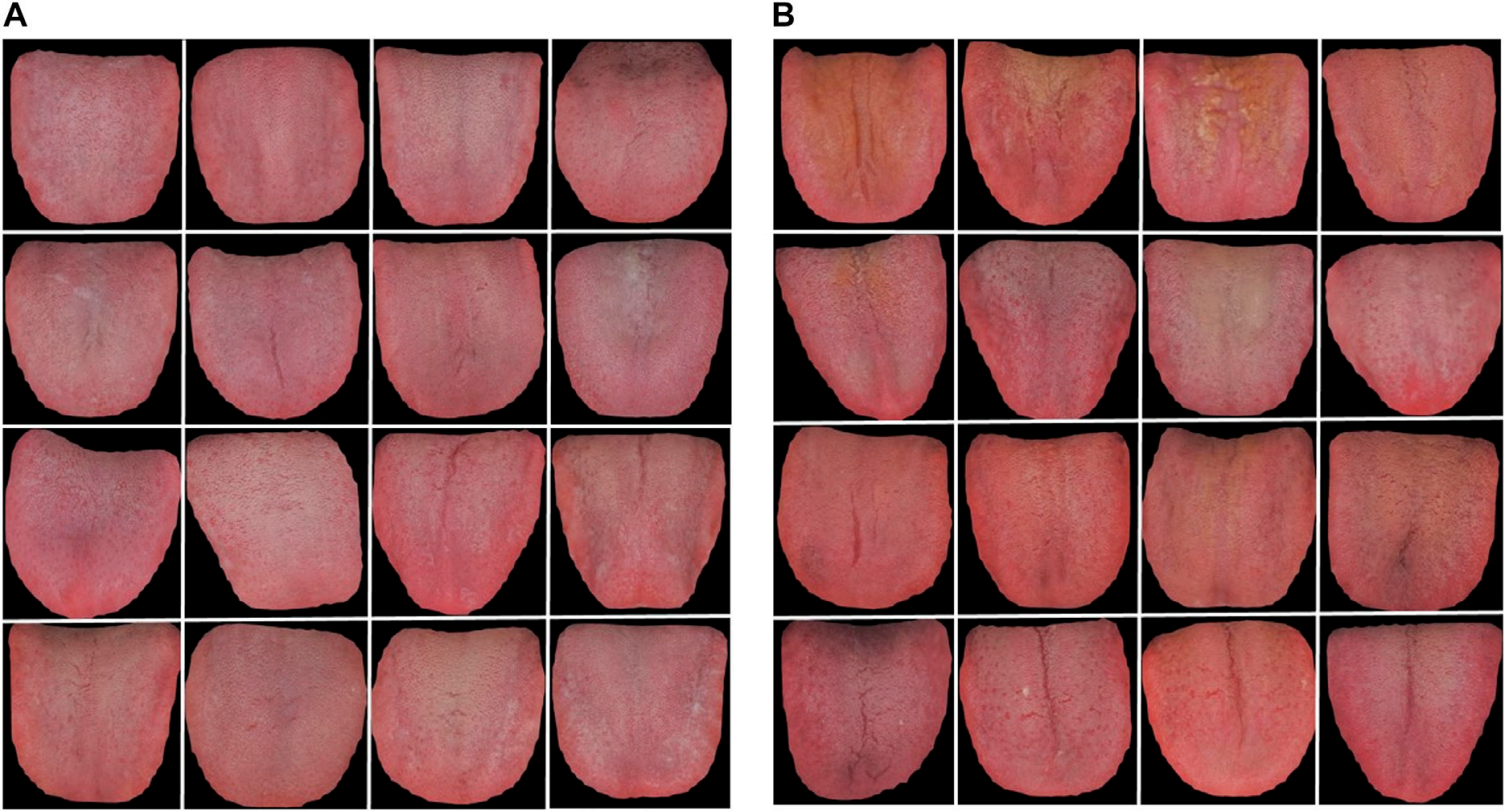
Representative tongue images of benign pulmonary nodules and lung cancer patients. **(A)** benign pulmonary nodules group, **(B)** lung cancer group.

In addition, to directly observe and understand the differences in the tongue images between the benign pulmonary nodule and lung cancer groups, representative tongue images of the two populations were shown in Figure 15.

## 4 Discussion

Early-stage lung cancer is a symptomless disease characterized mostly by pulmonary nodules, observable pathological products with a high clinical detection rate. Because it is challenging to determine whether they are benign or malignant, they are a focus of both domestic and international studies. According to TCM, most of its pathogenesis derives from a deficiency of vital qi and the interjunction of phlegm and blood stasis, and some pulmonary nodules can gradually enlarge and deteriorate. Such pulmonary nodules are actually the embryonic form of lung cancer, a very harmful disease among humans. Careful follow-up, observation of the direction of development and appropriate and timely treatment are needed. Determining whether pulmonary nodules are lung cancer is a serious medical issue. The accurate differentiation of benign and malignant nodules aids in early lung cancer detection, diagnosis, and treatment (Aberle et al., 2011; McWilliams et al., 2013). The likelihood of malignancy in pulmonary nodules being may be efficiently predicted, screening costs and the risk of morbidity and death can be decreased, and clinical decision-making can be supported by an accurate and useful model.

L stands for relative lightness, and Y stands for luminance; the lower the L and Y values are, the darker the color of the image. Statistical analysis of the tongue images of the three groups revealed that the lung cancer group’s TB-L, TC-L, TB-Y, and TC-Y values were lower than those in the pulmonary nodules group (*p* ≤ 0.01), indicating that the lung cancer group’s tongue image was darker and the brightness was lower. “a” represents the green‒red axis, and a positive value represents red. Cr reflects the difference between the red signal and the brightness value; the higher the Cr value is, the more reddish the tongue is. B represents the blue component; the higher the B value is, the bluer the tongue is; in other words, the concentration of blue components increases, and the tongue appears blue or purple. The lung cancer group’s TB-a, TC-a, TB-Cr, and TC-Cr values were higher than those in the benign pulmonary nodules group (*p* ≤ 0.01), while the TB-B, TC-B, TB-Cb, and TC-Cb values were lower (*p* ≤ 0.01), indicating that the tongues in the lung cancer group were more reddish and purple, and the tongues in the benign pulmonary nodules group were more cyanotic. “b” is the yellow‒blue color of the object, and positive values represent yellow. The lung cancer group’s TB-b and TC-b were higher than those in the benign pulmonary nodules group (*p* ≤ 0.01), indicating that the tongue coating in the lung cancer group was more yellow. In addition, the TC-L, TB-Y and TC-Y values in the benign pulmonary nodules group were significantly higher than those in the healthy controls (*p* ≤ 0.01), indicating that the tongue images of the former were brighter, while the TB-a, TC-a, TB-Cr and TC-Cr in the benign nodules group were lower than those in the healthy controls (*p* ≤ 0.01). The TB-G, TC-G, TB-B, TC-B, TB-Cb, and TC-Cb values were all higher than those in the healthy controls (*p* ≤ 0.01), indicating that the tongue images of patients in the benign pulmonary nodules group were paler and bluer than those of healthy controls. PerAll is the ratio of the tongue coating pixels to the total tongue area, and perPart is the ratio of tongue coating pixels to the area occupied by the tongue coating. The perAll and perPart values of the lung cancer patients were lower than those of the benign pulmonary nodules group (*p* ≤ 0.01), indicating that the lung cancer group had a smaller or nonexistent or thinner tongue coating area that was more likely to peel. The reason may be that lung cancer patients mostly have yin deficiency and fire prosperous syndrome, and their tongue images are characterized by a red tongue body and little and thin coating. In contrast, most benign pulmonary nodule patients have excess syndrome because the phlegm-dampness condenses, and the tongue coating was thick and greasy. The study’s findings concur with the TCM theory. The perAll value of the benign nodules group was higher than that of the healthy control group (*p* ≤ 0.01), indicating that the tongue coating area of patients with pulmonary nodules was higher than that of healthy controls. In addition, among the tongue texture indexes, a smaller CON, ENT and MEAN and a larger ASM indicate finer tongue texture, while for the tongue coating, these values indicate increased greasiness. In this study, TC-ASM was higher in the lung cancer group than in the benign pulmonary nodules group (*p* ≤ 0.05, *p* ≤ 0.01), and the other texture indexes (CON, ENT, MEAN) were not noticeably different, indicating that in the lung cancer group, the shallower tongue groove was associated with a less clear tongue texture and a greasier tongue coating. In conclusion, the research showed that there were differences in the correlation of tongue indexes among the three groups, and the level of correlation between the three groups had a specific pattern of distribution that could be used as the basis for categorizing various populations. In the future, the differences between tongue indexes of benign pulmonary nodules and lung cancer populations can be further explored based on multi-center and large samples in order to better support the intelligent classification of benign pulmonary nodules and lung cancer.

The diagnosis and management of pulmonary nodules depend greatly on the evaluation of benign and malignant conditions. Primary nodule malignancy probability prediction models have become increasingly popular in China in recent years (Schultz et al., 2008; Deppen and Grogan, 2015). Studies have been conducted on the prediction models of pulmonary nodules malignancy probability, including foreign models such as the Mayo Model (Swensen et al., 1997), Herder model (Herder et al., 2005; Vayntrub et al., 2021), Brock model (McWilliams et al., 2013; Kim et al., 2021), and VA model (Tanner et al., 2020). A risk prediction model combining clinical, blood, and imaging biomarkers can improve the noninvasive diagnosis of patients with indeterminate pulmonary nodules, potentially reducing the incidence of unnecessary invasive procedures and shortening the time to diagnosis (Kammer et al., 2021). Logistic regression is a popular supervised machine learning technique. The use of logistic regression has several advantages, including the ability to directly model classification probability without making any assumptions about the data distribution. In addition to categories, logistic regression can produce approximate probability predictions, which is very useful for many tasks where probability is required to support decision-making (Bucur et al., 2017; Hercus and Hudaib, 2020; Schober and Vetter, 2021a). According to this study’s modeling findings, it is possible to diagnose benign pulmonary nodules and lung cancer to a certain extent using objective tongue imaging data. Given that age differs between patients with pulmonary nodules and those with lung cancer and is the most frequently used baseline information, it was included in the modeling data set, and the modeling results showed that the classification efficiency was improved, suggesting that we can combine objective tongue image data with baseline data to create a better classification model.

This study still had some limitations. First, the sample size of this study was small, future research still require multi-center studies with larger samples. Secondly, this study was only based on tongue data for lung cancer risk warning, and the model accuracy was not high. It might not be sufficient to be applied to cancer screening in real world. In the future, pulse data, face data and Western medical index can be further integrated, using multi-modal data fusion technology to create a lung cancer risk warning model based on multi-dimensional data that is more accurate and more suited for clinical practical applications.

## 5 Conclusion

The objective tongue image data of benign pulmonary nodules and lung cancer patients had different statistical characteristics and correlations. TB-L, TB-a, TB-b, TB-ASM, TC-L, TC-a, TC-b, TC-ASM, perAll, and perPart played an important role in the differential diagnosis of benign pulmonary nodules and lung cancer. The performance of models built on tongue image and baseline data outperformed models built only on tongue image or baseline data. Adding objective tongue image data to baseline data can significantly improve the efficacy of lung cancer prediction models.

## Data Availability

The data analyzed in this study is subject to the following licenses/restrictions: The datasets generated and analyzed during the current study are not publicly available due to the confidentiality of the data, which is an important component of the National Key Technology R&D Program of the 13th Five-Year Plan (no. 2017YFC1703301) in China but are available from the corresponding author on reasonable request. Requests to access these datasets should be directed to YS, yilinlife94@126.com.

## Supplementary material

Supplementary Figure S1 Correlation coefficient values of the tongue image data for healthy controls.

Supplementary Figure S2 Correlation coefficient values of the tongue image data for benign pulmonary nodules.

Supplementary Figure S3 Correlation coefficient values of the tongue image data for lung cancar group.

Supplementary Figure S4 The precision-recall curves of models based on baseline.

Supplementary Figure S5 The precision-recall curves of models based on tongue image data.

Supplementary Figure S6 The precision-recall curves of models based on baseline and tongue image data.
