# Supplementary figures and images for: A lung cancer risk warning model based on tongue images
Source: Front Physiol. 2023 Jun 1;14:1154294. doi: 10.3389/fphys.2023.1154294 (PMC10267397; doi:10.3389/fphys.2023.1154294)

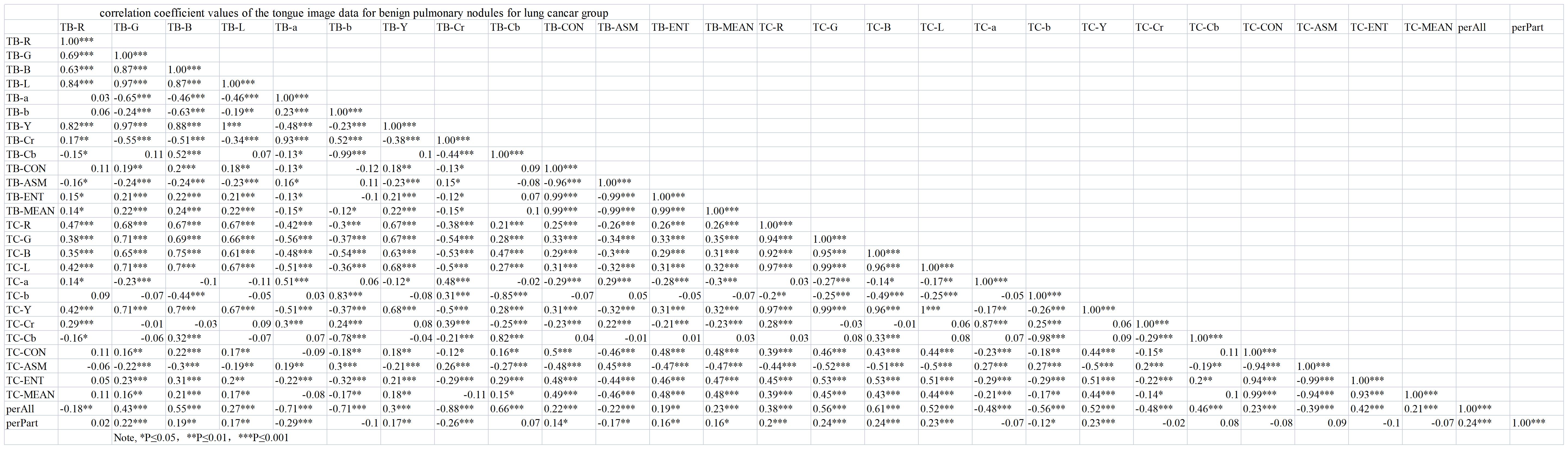

Supplement: Supplementary file 1 [file Image3.jpg]

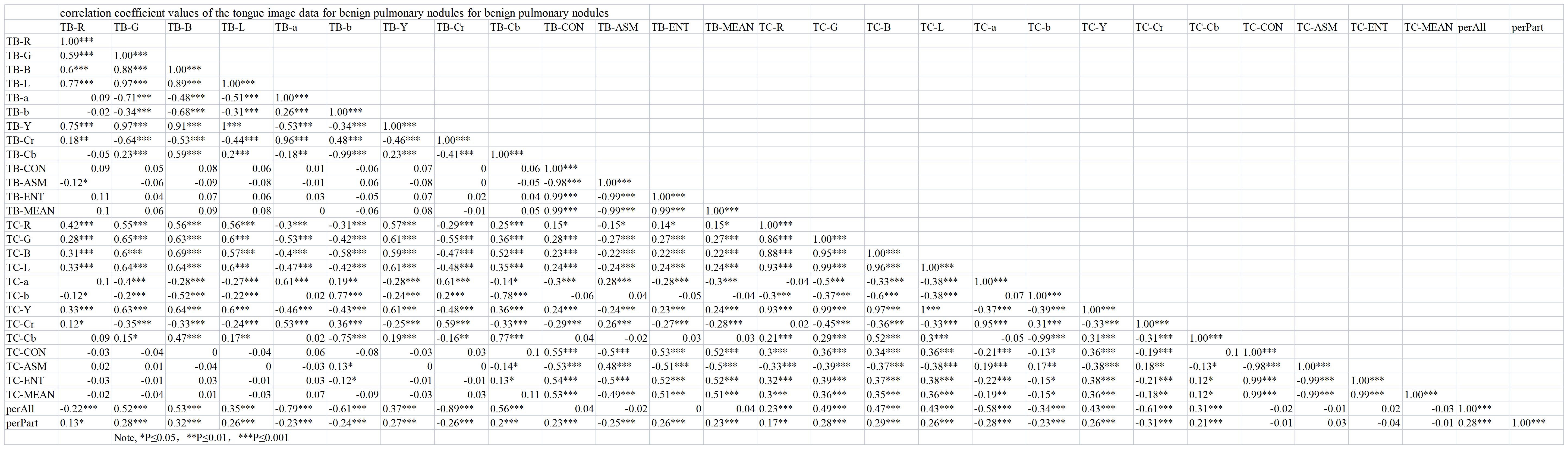

Supplement: Supplementary file 2 [file Image2.jpg]

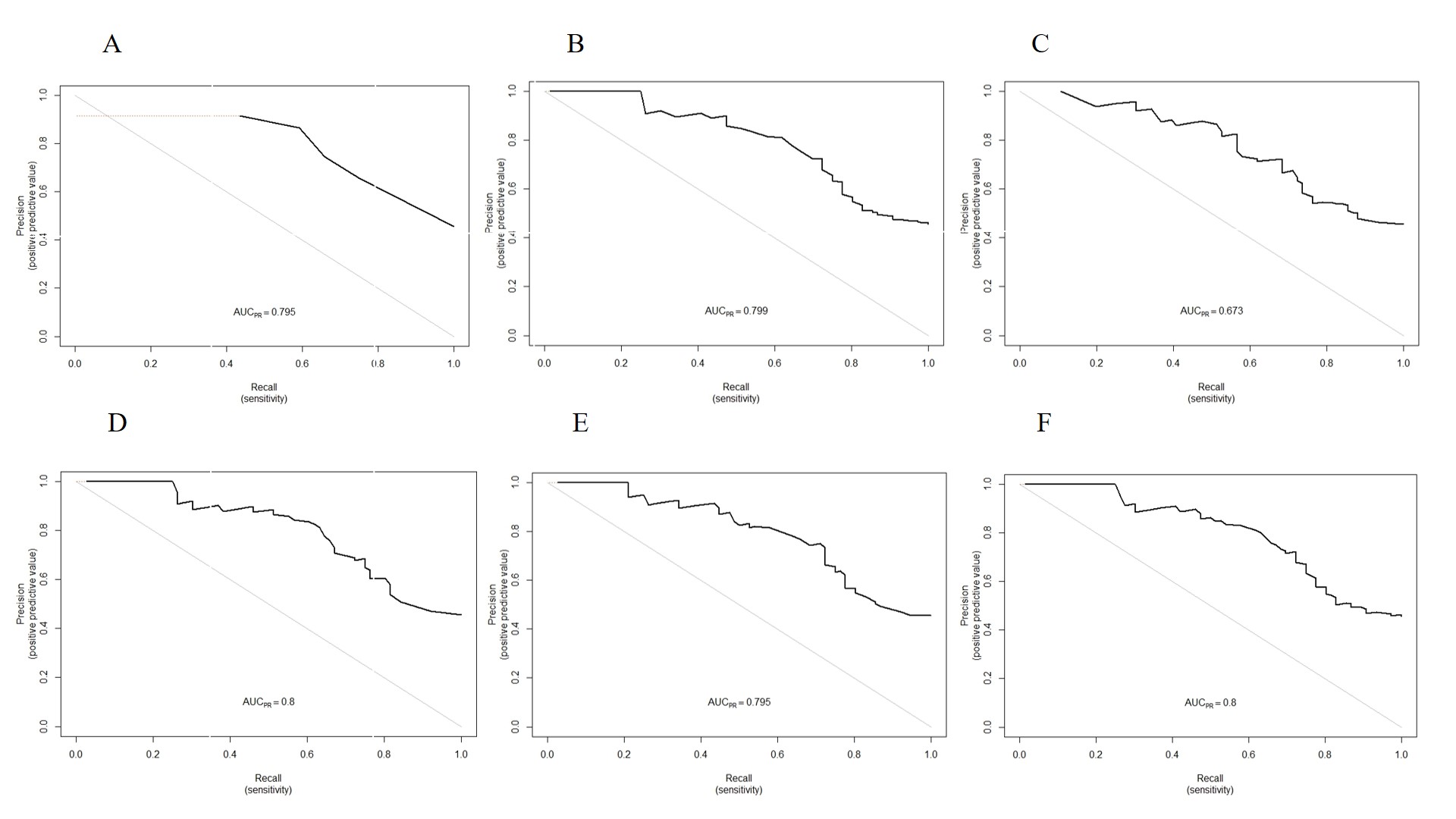

Supplement: Supplementary file 3 [file Image4.JPEG]

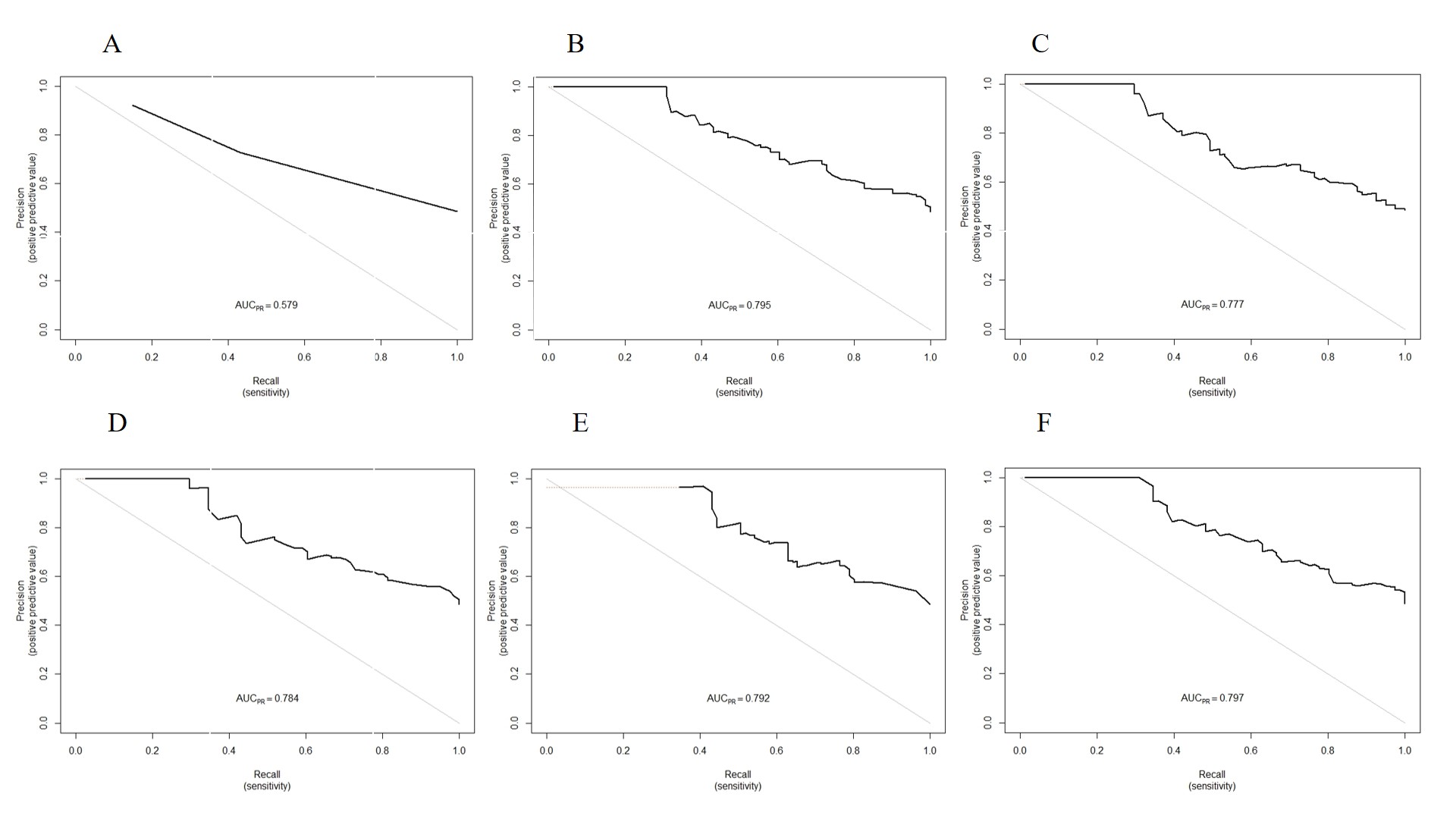

Supplement: Supplementary file 4 [file Image5.JPEG]

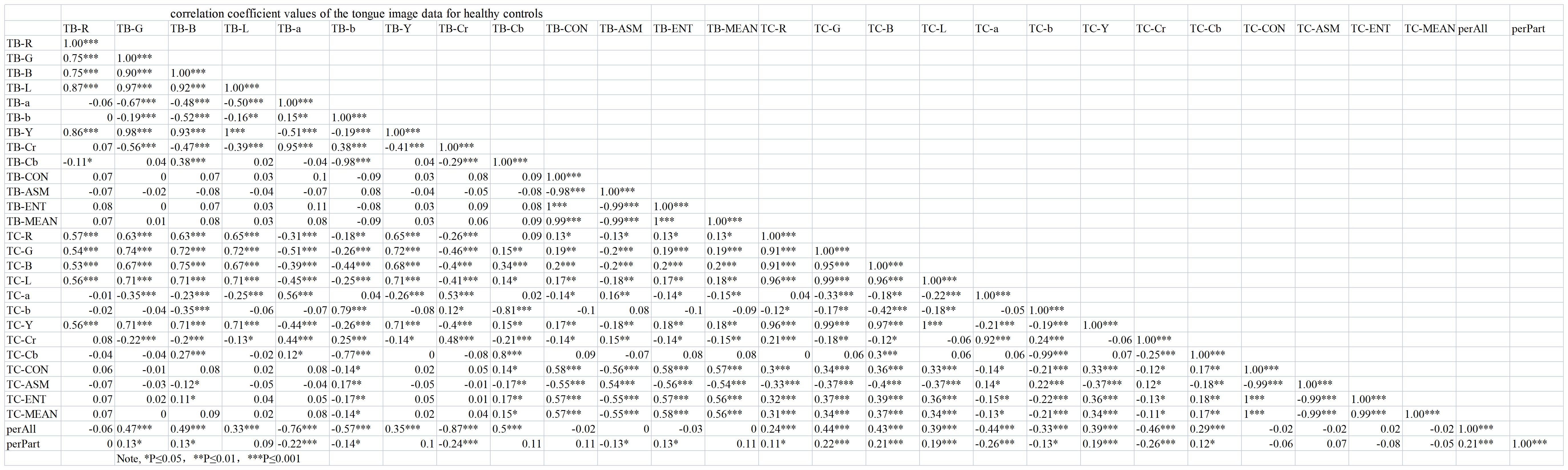

Supplement: Supplementary file 6 [file Image1.jpg]

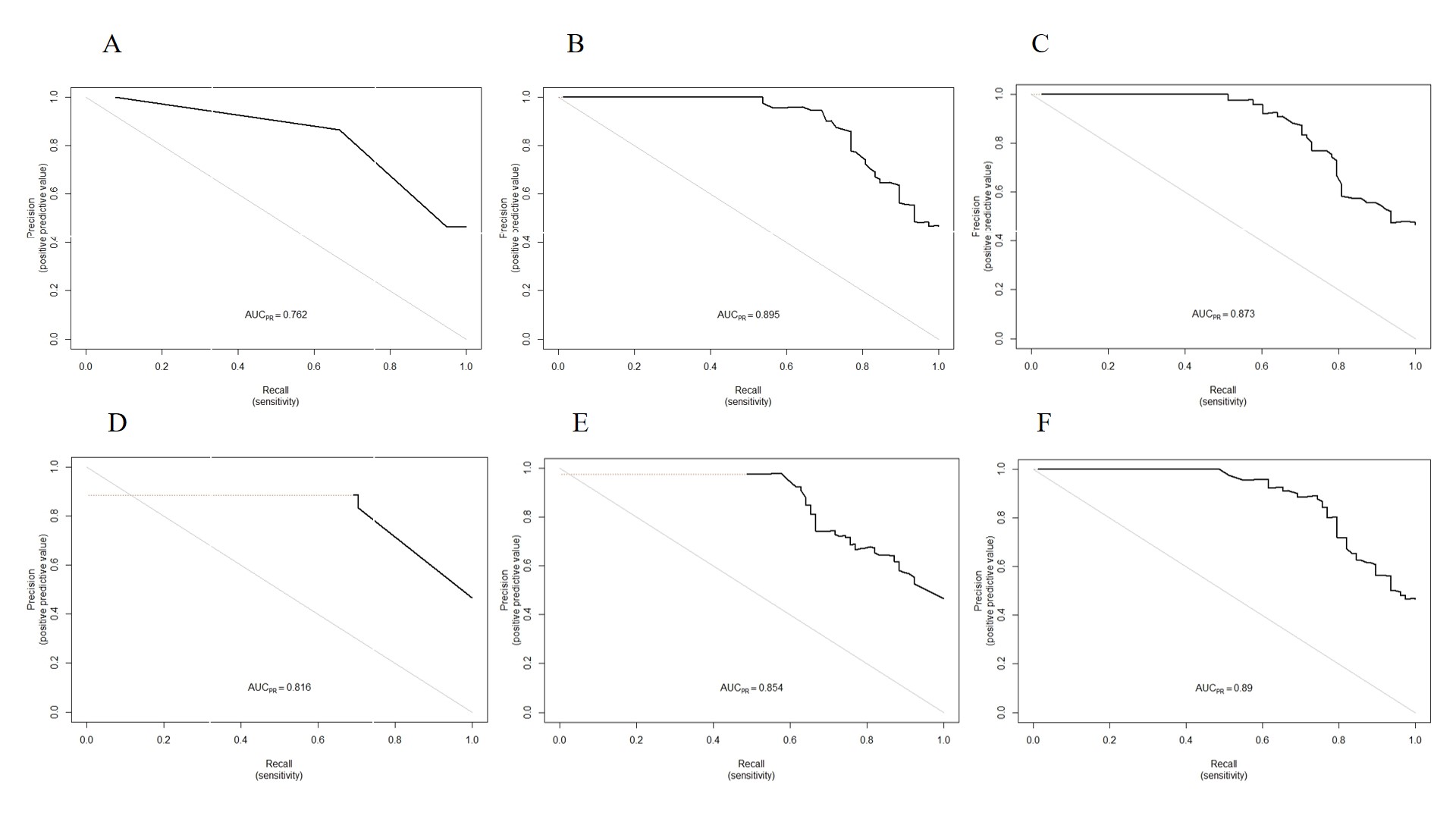

Supplement: Supplementary file 7 [file Image6.JPEG]
